# Is Middle-Upper Arm Circumference “normally” distributed? Secondary data analysis of 852 nutrition surveys

**DOI:** 10.1186/s12982-016-0048-9

**Published:** 2016-05-04

**Authors:** Severine Frison, Francesco Checchi, Marko Kerac, Jennifer Nicholas

**Affiliations:** Department of Population Health, London School of Hygiene and Tropical Medicine, Keppel Street, London, WC1E 7HT UK; Department of Medical Statistics, London School of Hygiene and Tropical Medicine, Keppel Street, London, WC1E 7HT UK; Faculty of Public Health and Policy, LSHTM and Humanitarian Technical Unit, Save the Children, 207 Old Street, London, EC1V 9NR UK

**Keywords:** Normal distribution, Middle-Upper Arm Circumference, Child malnutrition, Wasting, Probit

## Abstract

**Background:**

Wasting is a major public health issue throughout the developing world. Out of the 6.9 million estimated deaths among children under five annually, over 800,000 deaths (11.6 %) are attributed to wasting. Wasting is quantified as low Weight-For-Height (WFH) and/or low Mid-Upper Arm Circumference (MUAC) (since 2005). Many statistical procedures are based on the assumption that the data used are normally distributed. Analyses have been conducted on the distribution of WFH but there are no equivalent studies on the distribution of MUAC.

**Methods:**

This secondary data analysis assesses the normality of the MUAC distributions of 852 nutrition cross-sectional survey datasets of children from 6 to 59 months old and examines different approaches to normalise “non-normal” distributions.

**Results:**

The distribution of MUAC showed no departure from a normal distribution in 319 (37.7 %) distributions using the Shapiro–Wilk test. Out of the 533 surveys showing departure from a normal distribution, 183 (34.3 %) were skewed (D’Agostino test) and 196 (36.8 %) had a kurtosis different to the one observed in the normal distribution (Anscombe–Glynn test). Testing for normality can be sensitive to data quality, design effect and sample size. Out of the 533 surveys showing departure from a normal distribution, 294 (55.2 %) showed high digit preference, 164 (30.8 %) had a large design effect, and 204 (38.3 %) a large sample size. Spline and LOESS smoothing techniques were explored and both techniques work well. After Spline smoothing, 56.7 % of the MUAC distributions showing departure from normality were “normalised” and 59.7 % after LOESS. Box-Cox power transformation had similar results on distributions showing departure from normality with 57 % of distributions approximating “normal” after transformation. Applying Box-Cox transformation after Spline or Loess smoothing techniques increased that proportion to 82.4 and 82.7 % respectively.

**Conclusion:**

This suggests that statistical approaches relying on the normal distribution assumption can be successfully applied to MUAC. In light of this promising finding, further research is ongoing to evaluate the performance of a normal distribution based approach to estimating the prevalence of wasting using MUAC.

## Background

Wasting is a major public health issue throughout the developing world. The United Nations Children’s Fund’s (UNICEF) latest report on the State of the World’s Children [1] estimates that 10 % of children under 5 years old in least developed countries are wasted. Out of the 6.9 million estimated deaths among children under five annually, over 800,000 deaths (12.6 %) are attributed to wasting [2]. Wasting is quantified as Weight-For-Height (WFH) < −2 standard deviations (SD) from the World Health Organization (WHO) reference median and/or Mid-Upper Arm Circumference (MUAC) < 125 mm. MUAC has been adopted by the World Health Organisation (WHO) as a measure of wasting and is increasingly recognised as a very useful measure of anthropometric status [3, 4].

Many statistical procedures are based on the assumption that the data follow a normal distribution. The shape of the normal distribution (the characteristic “bell curve”) is quantified by two parameters: the mean and the standard deviation, and follows important properties: (1) it is always symmetrical with equal areas on both sides of the curve; (2) the highest point on the curve corresponds to the mean which equals the median and the mode; (3) the spread of the curve is determined by the standard deviation; and (4) as with all probability density functions the area under the curve must sum to the total probability of 1 [5]. The distribution of many characteristics in nature is normal or follows some form that can be derived from the normal distribution and specific statistical approaches are based on the properties of a normal distribution. For example, the probit approach [5, 6] estimates the prevalence of wasting as the cumulative probability of lying below the relevant MUAC cut-point based on the mean and standard deviation (SD) of the observed data [5, 6].

There are graphical and statistical methods for evaluating normality. Graphical methods include histograms and normality plots. Statistical methods include diagnostic hypothesis tests for normality, and a normal distribution has a skewness of 0 and kurtosis of 3 [7, 8]. Skewness is a measure of the asymmetry of a distribution around its mean while Kurtosis indicates heavy tails and “peakedness” relative to a normal distribution [9, 10]. The ability to detect departure from a normal distribution can be sensitive to local peaks and troughs in the distribution. A way to deal successfully with this issue is to apply smoothing techniques (fit a smooth curve to a set of noisy observations) using different methods such Spline function or Locally Weighted Scatterplot Smoothing (LOESS) [11–13]. For distribution originating from cluster surveys, it may be expected that high clustering in observations (large design effect) lead to asymmetric distributions, e.g. featuring a long tail of low MUAC observations. When a variable is not normally distributed for a reason other than the ones above, it can often be transformed and tested for normality using power transformations such as the Box-Cox transformation [14, 15].

Although the violation of the normal distribution assumption often increases chances of committing either a type I or II error, very few researchers test whether the assumption does indeed hold before carrying out statistical analyses [16, 17]. Previous studies have assessed the distribution of WFH [18–20] but there are no equivalent studies on the distribution of MUAC. This paper assesses the normality of the MUAC distribution graphically and statistically, and explores different transformations and smoothing techniques in order to reach normality. Findings presented pertain to a broader project to develop a more efficient method for estimating the prevalence of wasting using MUAC as the primary index, which relies heavily on MUAC distributions meeting normality criteria.

## Methods

### Study design and inclusion criteria

A total of 1068 cross-sectional survey datasets from various settings were shared by six organisations (UNICEF, Food Security and Nutrition Analysis Unit, Epicentre/Médecins Sans Frontières, Action Against Hunger, Concern Worldwide and Goal). The study size depended on availability of surveys and on specific inclusion criteria. Eligible datasets had to: (1) include MUAC, oedema, age, weight and height as well as meta-data on country, livelihood, residence, cluster (if cluster surveys) and date; (2) have a minimum of 25 clusters if cluster surveys [21, 22]. The last criteria aimed to minimise selection bias, as surveys with a small number of clusters may not be representative of the population. The surveys were exhaustive or clustered surveys. The datasets were cleaned and records with extreme or missing values were excluded: Children were excluded if any of the following data were missing: age; sex; height; weight; MUAC; oedema. Those with highly improbable extreme values (‘flags’) were also excluded from analysis: MUAC < 85 mm or MUAC > 200 mm, age < 6 months or age > 59 months, Weight-For-Age (WFA) < −6.0 SD or WFA > +5.0 SD, Height-For-Age (HFA) < −6.0 SD or HFA > +6.0 SD, WFH < −5.0 SD or WFH > +5.0 SD (WHO “flags” were applied on SD for WFH, WFA and HFA [23]).

### Database

Out of the 1068 surveys collected, 852 surveys were included in the secondary data analysis (55 exhaustive surveys and 797 clustered surveys). The 852 surveys contained 668,975 children of which 25,134 (3.76 %) presented highly improbable values and were excluded from the analysis. The database included six variables for anthropometry (sex, MUAC, oedema, age, weight and height), six meta-data variables (organisation, country, livelihood, residence, cluster (when cluster surveys) and date). Other variables were computed for the purpose of this analysis: (1) the normality of the distribution (binary: 1 = yes/0 = no using Shapiro–Wilk test), (2) the skewness and Kurtosis of MUAC as continuous and binary (binary: 1 = yes/0 = no whether the data was skewed or peaked using D’Agostino and Anscombe–Glynn tests respectively), (3) the design effect of surveys (large over 3) (4) digit preference of MUAC. The digit preference variable was equal to 1—absolute (0.1-proportion of each digit preference). Assuming that the proportion of measurements ending with 0, 1, 2, 3, 4, 5, 6, 7, 8, 9 should equal 10 % and therefore that the highest score was 1, the lesser the digit preference, the higher the score. A score equal or over 0.75 corresponded to a low digit preference, and under 0.75 to a high digit preference, and (5) survey size category (large size over 900).

### Data analysis

The normality of the MUAC distributions was assessed graphically looking at histograms of MUAC distributions and Q–Q plots (probability plot, “Q” stands for quantile). Q–Q plots show sorted values from the data set against the expected values of the corresponding quantiles from the standard normal distribution. The measure of departure from normality was also investigated statistically through Shapiro–Wilk test as well as the D’Agostino test to assess the skewedness and Anscombe–Glynn test to assess the peakedness of MUAC distributions. For each statistical test, a p value less than 0.05 indicates evidence for departure from a normal distribution.

Different methods were explored to transform non-normal distributions into normal: (1) Spline smoothing (using a spline function) and LOESS (locally weighted scatterplot smoothing using local polynomial regression fitting) techniques were applied to all distribution showing departure from a normal distribution (Shapiro–Wilk test). While smoothing the data, three criteria were applied: the mean MUAC and MUAC SD, after back-transformation of the smoothed data must be almost unchanged from the non-smoothed mean and SD (properties of a normal distribution is defined by the mean and the SD), and the Shapiro–Wilk test *p* value has to exceed 0.05. (2) Box-Cox power transformation was applied to all survey showing departure from a normal distribution, and (3) Box-Cox power transformation was applied on surveys showing departure from “normality” after smoothing techniques had been applied.

Spline smoothing fits a spline with knots at every data point (x) by estimating its parameters minimizing the usual sum of squares plus a roughness penalty (λ). If λ → 0 imposes no penalty (very close fit), but the resulting curve could be very noisy as it follows every detail in the data. As λ → ∞ the penalty dominates and the solution converges to the ordinary least square line. LOESS is a fairly direct generalization of traditional least-squares methods for data analysis. It fits a polynomial surface determined by one or more numerical predictors, using local fitting. That is, for the fit at point *x*, the fit is made using points in a neighbourhood of *x*, weighted by their distance from *x* (with differences in ‘parametric’ variables being ignored when computing the distance). The size of the neighbourhood is controlled by *α* (set by span).

The Box-Cox method transforms data into a “normal” shape using parameter λ corresponding to different transformations (i.e. λ = 1.00: no transformation needed; λ = 0.50: square root transformation λ = 0.29: for a transforming power between cube and fourth root λ = 0.33: cube root transformation λ = 0.25: fourth root transformation λ = 0.00: natural log transformation λ = −0.50: reciprocal square root transformation λ = −1.00: reciprocal (inverse) transformation and so forth). The most appropriate value of λ was identified as that which minimised the departure from a normal distribution on the Shapiro–Wilk test.

R studio and STATA 13 were used for all analyses [24, 25].

Ethics approval for the project was sought and obtained from the Ethics Committee of the London School of Hygiene and Tropical Medicine (LSHTM Ethics reference 6158).

## Results

The distribution of MUAC showed no departure from a normal distribution in 37.4 % (319 out of 852) of the MUAC distributions using the Shapiro–Wilk test. Out of the 533 surveys showing departure from a normal distribution, 183 (34.3 %) were skewed (D’Agostino test), 196 (36.8 %) had a kurtosis different to the one observed in the normal distribution (Anscombe–Glynn test) and 70 (13.1 %) showed both features. The sensitivity of the Shapiro–Wilk test to departure from normality is influenced by the presence of local peaks and troughs in the distribution such as those caused by digit preference (poor data quality), the design effect (high design effect may lead to asymmetric distributions), and sample size (large sample size results in greater power to detect small departures from a normal distribution). Out of the 533 surveys showing departure from normal distribution, 294 (55.2 %) showed high digit preference (score < 0.75) 164 (30.8 %) had a large design effect (over 3), and 204 (38.3 %) a large sample size (>900) (Table 1). The skewness and kurtosis of surveys showing departure from normality included values above and below the value for a normal distribution (0 for skewedness and 3 for kurtosis) indicating surveys skewed to right as well as to the left and survey with a distribution flatter or more peaked than the normal distribution (Table 2).

**Table 1:** Characteristics of surveys showing departure from a normal distribution (Shapiro–Wilk test, p < 0.05) and effect of transformation and smoothing on specific characteristics (N = 533)

Surveys failing Shapiro–Wilk test (p < 0.05) N = 533 (62.6 %)	N (%)	N (%) with “normal” distribution after transformation or smoothing		
		Box-Cox	Spline	Loess
All surveys^a^	533 (100)	301 (56.5)	318 (59.7)	304 (57.0)
By key survey characteristics				
Skewed^b^	183 (34.3)	113 (61.7)	81 (49.4)	89 (48.6)
Non-normal kurtosis^c^	196 (36.8)	62 (31.6)	137 (69.9)	139 (70.9)
Skewed and non-normal kurtosis^b, c^	70 (13.1)	23(32.9)	41 (58.6)	43 (61.4)
Large design effect (>3)	164 (30.8)	86 (52.4)	81 (49.4)	92 (56.1)
High digit preference (score < 0.75)	294 (55.2)	143 (48.6)	170 (57.8)	178 (60.6)
Large sample size (n > 900)	204 (38.3)	122 (59.8)	95 (46.6)	101 (49.5)

^a^Shapiro–Wilk test (p < 0.05); ^b^ D’Agostino test (p < 0.05); ^c^ Anscombe–Glynn test (p < 0.05)

**Table 2:** Skewness and kurtosis of survey showing departure from a normal distribution (n = 533)

	Minimum	Lower quartile	Median	Mean	Upper quartile	Maximum
Skewness	−0.61	−0.15	−0.01	−0.01	0.11	0.91
Kurtosis	2.26	2.3	3.2	3.24	3.45	5.27

**Fig. 1:**
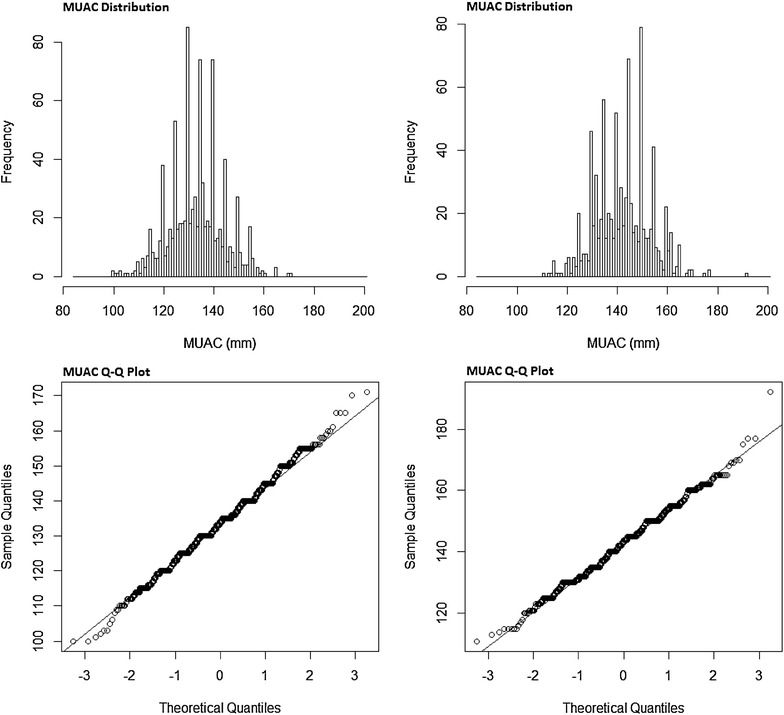
Examples of non-normal (Shapiro–Wilk test) MUAC distributions and their respective Q–Q plot

Figure 1 shows examples of distributions of MUAC and their respective Q–Q plots for two surveys with very “non-normal” distribution (very low p-value Shapiro–Wilk test) but skewness and kurtosis close to those observed in a normal distribution. Visually neither of the distribution seemed skewed or peaked but digit preferences were visible in both cases which suggest this might be the reason behind the low p-value (Shapiro–Wilk test).

**Fig. 2:**
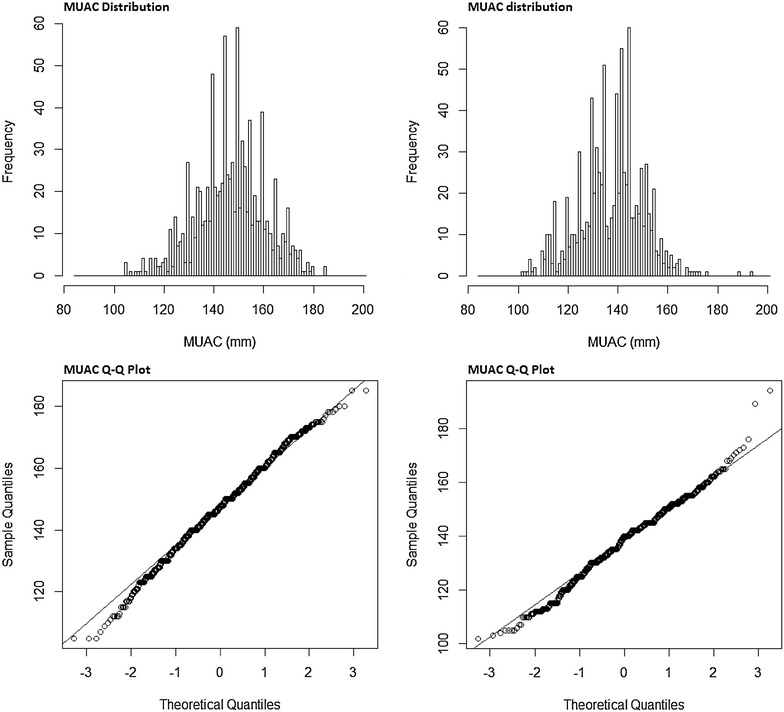
Examples of a skewed and a peaked distribution and their respective Q–Q plots (D’Agostino and Anscombe–Glynn tests respectively)

Figure 2 shows examples of distributions of MUAC and their respective Q–Q plots for two surveys with “non-normal” distribution (low p-value Shapiro–Wilk test) and also skew or kurtosis different to that observed in the normal distribution. The distribution and Q–Q-Plot for the survey shown in panel A has skewed distribution (D’Agostino test) and that shown in panel B has peaked distribution (Anscombe–Glynn test). The distribution in panel A was slightly skewed to the left and there were very visible digit preferences. Peaks are visible in the distribution in panel B as well as digit preferences.

Table 1 as well as Figs. 1 and 2 suggest the main reason for departure from a normal distribution is due to local peaks and troughs.

### Smoothing techniques

Spline and LOESS smoothing techniques were explored and both techniques work well. After applying Spline smoothing to the distributions showing departure from normality, 301 (56.5 %) of the MUAC distributions showed no departure from normality and 318 (59.7 %) after LOESS (Table 3).

**Table 3:** Smoothing and transformation of surveys showing departure from a normal distribution (n = 533)

Type of transformation or smoothing technique applied to “non-normal” distributions (n = 533)		N (%) “normal” distributions
Smoothing	Spline	301 (56.5)
	Loess	318 (59.7)
Box-Cox transformation	Power transformations	304 (57.0)
Smoothing and Box-Cox transformation	Box-Cox after Spline	439 (82.4)
	Box-Cox after Loess	441 (82.7)

The average mean MUAC change after Spline smoothing was 0.1 and the mean SD MUAC change was 0.8. All surveys had an average mean MUAC change under 10 and 90 % had a SD change under 10 %. After LOESS smoothing, the average mean MUAC change was 0.2 and the average SD MUAC change was 0.9. All surveys had a mean MUAC change under 10 and 84 % had a SD change under 10 %.

The effect of Spline and Loess smoothing on “non-normal” distributions with large design effect, high digit preference, large sample size as well as on skewed distributions and distributions with a kurtosis different from a normal distribution (flat or peaked) was considerable. Approximately half of surveys with large design effect were normalised after Spline and LOESS (49.4 and 56.1 % respectively), about two-third of surveys with high digit preference had a distribution approximating normal after Spline and LOESS (60.6 and 57.8 % respectively), half of surveys with large sample size (46.6 and 49.54 % respectively) as well as half of skewed distributions (49.4 and 48.6 % respectively) and over two-third of surveys with kurtosis different from normal were approximating a normal distribution after Spline and Loess smoothing (69.9 and 70.9 % respectively) (Table 3).

### Box-Cox power transformation

Power transformations are typically used to “normalise” skewed distributions. Common power transformations include log, reciprocal, square and square root transformations. After applying the Box-Cox transformation to the 533 distributions showing departure from normality, 304 (57 %) of the distribution were converted to “normal” (Table 3).

The summary statistics of the Box-Cox transformation coefficient [Lambda (λ)] suggest that a variety of different power transformations were required for different surveys and few Lambda values corresponded to common power transformations (Table 4).

**Table 4:** Summary statistics of the Box-Cox transformation coefficient (Lamdba) for surveys showing departure from normality (n = 533)

	Minimum	Lower quartile	Median	Mean	Upper quartile	Maximum
Lambda (λ)	−1.2	0.61	1.08	1.03	1.51	2.73

The effect of Box-Cox transformation on skewed distributions was sizable with almost two-third of skewed distribution approximation a normal distribution after Bo-Cox transformation (61.7 %). About half of surveys with large design effect, high digit preference and large sample size distribution were approximating a normal distribution after Box-Cox (52.4, 48.6 and 59.8 % respectively). The effect on distributions with a kurtosis different from normal was less marked with a third (31.6 %) approximation a normal distribution after Box-Cox transformation (Table 3).

### Smoothing and Box-Cox transformation

Applying Box-Cox transformation on surveys showing departure from a normal distribution after Loess or smoothing techniques increased further the number of “normal” distributions with 401 distributions (82.7 %) after Loess and Box-Cox and 439 (82.4 %) after Spline and Box-Cox (Table 3).

## Discussion

Over a third of MUAC distributions showed no departure from normality without any transformation and three quarters showed no departure once the data were smoothed or after Box-cox transformation. Applying Box-Cox transformation on surveys showing departure from normality after smoothing resulted in over 80 % of surveys approximating a normal distribution.

Loess smoothing had slightly better outcome then Spline smoothing or Box-Cox transformation alone in terms of number of distributions approximating a normal distribution but had a change in mean and SD slightly higher (but acceptable) than Spline smoothing. Although Box-Cox transformation performed well, data transformations change the nature of the variable, and any Lambda (λ) less than 0.00 has the effect of reversing the order of the data. Even though back transformation restores the data, care should be taken when applying this function [15].

The normality of MUAC distributions is affected by sample size, high digit preference, kurtosis different than a normal distribution and skewness. Datasets with larger sample size increase the power of the test to detect small differences when applying normality tests. Digit preference reflects the quality of the data. Training measurers to increase accuracy and precision would decrease digit preference. Both effects were lessened (two-third for digit preference and half for sample size) applying smoothing techniques to the distributions as well as applying Box-Cox transformation (half for both high digit preference and large sample size surveys). Although a third of surveys showing departure from a normal distribution were skewed or had a kurtosis different from a normal distribution, half and over two-thirds (respectively) of these were “normalised” after smoothing. Box-Cox transformation was effective on skewed distributions (almost two-third of skewed distribution “normalised”) but didn’t perform as well on distributions with a kurtosis different from normal (a third of distributions approximated a normal distribution after Box-Cox).

Few studies have assessed the distribution of WFH. Two looked at the standard deviations of the WFH distributions. In 1977, Waterlow et al. [19]. showed that the WFH distributions were skewed at the upper centiles. Their analysis was performed on data from surveillance or surveys involving nutrition and anthropometry in young children up to the age of 10 years. In 2006, Mei et al. [18] analysed data from 51 DHS surveys representing 34 developing Countries. They found a mean WFH and SD WFH (z-scores) of 0.06 and 1.40 respectively. The mean ranged from −0.91 to 0.83 and the SD range from 1.03 to 1.55. They concluded that their analysis confirms the WHO assertion that the SD remains in a relatively small range (i.e. close to SD from a standard normal distribution), no matter the *Z*-score mean although the observed range of SD for was consistently wider. Finally, in 2013, Blanton and Bilukha showed that based on the Shapiro–Wilk test for normality, 6 surveys out of the 10 surveys included in their analysis were “non-normal”. All of the surveys had a small amount of skewness ranging from −0.17 and 0.31 as well as a relatively small amount of kurtosis ranging from 0.15 to 0.75.

Regarding the assessment of MUAC distributions, no equivalent studies were conducted. In 2013, data analysis from 560 cross sectional surveys conducted by Dale et al. [26]. mention the use of Box-Cox transformation to normalise MUAC and WFH data but do not give further details.

There is one main limitation to this study. The database was built based on available small scale surveys that were mainly conducted in areas where there was suspicion of a problem (i.e. high wasting prevalence) compared to national DHS and MICS surveys that are conducted every 3–5 years and show long term trends. However, we do not believe this affects the generalisability of the study. Future research might explore similar analysis on different datasets.

## Conclusions

Over a third of the MUAC distributions of our database were normally distributed. MUAC distributions can easily be normalised applying simple smoothing techniques if the distribution is noisy or displays digit preference and then Box-Cox transformation if indicated (i.e. if data is skewed). This suggests that statistical approaches relying on the normal distribution assumption can be successfully applied to MUAC. In light of this promising finding, further research is ongoing to evaluate the performance of a normal distribution based approach to estimating the prevalence of wasting using MUAC.

## Abbreviations

LOESS: Locally Weighted Scatterplot Smoothing
MUAC: Middle-Upper Arm Circumference
UNICEF: United Nations Children’s Fund’s
SD: standard deviation
WFH: Weight-For-Height
WHO: World Health Organization
